# Ameliorative Effects of 5-Hydroxymethyl-2-furfural (5-HMF) from *Schisandra chinensis* on Alcoholic Liver Oxidative Injury in Mice

**DOI:** 10.3390/ijms16022446

**Published:** 2015-01-22

**Authors:** Wei Li, Xin-Nan Qu, Ye Han, Si-Wen Zheng, Jia Wang, Ying-Ping Wang

**Affiliations:** 1 Institute of Special Wild Economic Animals and Plant, Chinese Academy of Agricultural Sciences, Changchun 132109, China; E-Mails: w.li@jlau.edu.cn (W.L.); siwen_z@yahoo.com (S.-W.Z.); wangjia@jlau.edu.cn (J.W.); 2 College of Chinese Medicinal Materials, Jilin Agricultural University, Changchun 130118, China; E-Mails: quxinnan@126.com (X.-N.Q.); hanye@jlau.edu.cn (Y.H.)

**Keywords:** 5-HMF, *Schisandra chinensis*, alcohol liver injury, oxidative stress

## Abstract

The aim of this paper is to evaluate the protective effect of 5-hydroxymethyl-2-furfural (5-HMF) on acute alcohol-induced liver oxidative injury in mice. 5-HMF, a maillard reaction product, was isolated from the fruits of *Schisandra chinensis* for animal experiments. Experimental ICR mice were pretreated with different doses of 5-HMF (7.5, 15, and 30 mg/kg) for seven days by gavage feeding. Biochemical markers and enzymatic antioxidants from serum and liver tissue were examined. Our results showed that the activities of ALT (alanine aminotransferase), AST (aspartate transaminase), TC (total cholesterol), TG (triglyceride), L-DLC (low density lipoprotein) in serum and the levels of MDA (malondialdehyde) in liver tissue, decreased significantly (*p <* 0.05) in the 5-HMF-treated group compared with the alcohol group. On the contrary, enzymatic antioxidants CAT (catalase), GSH-Px (glutathione peroxidase), and GSH SOD (superoxide dismutase) were markedly elevated in liver tissue treated with 5-HMF (*p <* 0.05). Furthermore, the hepatic levels of pro-inflammatory response marker tumor necrosis factor-alpha (TNF-α) and interleukin-1β (IL-1β) were significantly suppressed (*p <* 0.05). Histopathological examination revealed that 5-HMF (30 mg/kg) pretreatment noticeably prevented alcohol-induced hepatocyte apoptosis and fatty degeneration. It is suggested that the hepatoprotective effects exhibited by 5-HMF on alcohol-induced liver oxidative injury may be due to its potent antioxidant properties.

## 1. Introduction

Accumulating evidence indicates that long-term alcohol abuse and alcohol dependence leads to many diseases, such as malnutrition and alcoholic liver diseases (ALD) [1]. Generally, oxidative stress and lipid peroxidation are known to play an important role in the pathogenesis of ALD [2,3]. Since oxidative stress is involved the development of ALD, using the antioxidants would potentially blunt ethanol-induced oxidative stress and prevent pathogenesis [4]. Therefore, the predominant source of antioxidants and their role in preventing ethanol-induced liver injury is an important target.

**Figure 1:**
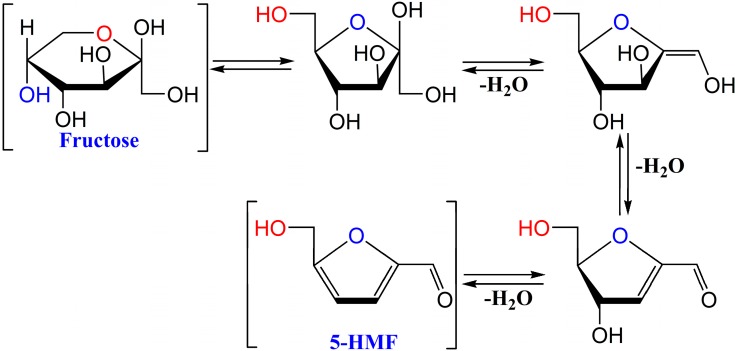
The production pathway of 5-HMF from fructose.

5-Hydroxymethylfurfural (5-HMF, CAS NO. 67-47-0), a product of the famous Maillard reaction, is mainly generated by acid-catalysed thermal dehydration of fructose and identified as a flavoring substance in a wide variety of heat-processed products (Figure 1) [5,6]. Interestingly, it is also found in large quantities in heat-processed Traditional Chinese Medicines such as *Lycium chinense* [7], *Cornus officinalis* [8], *Rehmanniae radix* [9], *Polygonum multiflorum* [10], and *Schisandra chinensis* [11]. In the last decades, there has been intense debate concerning 5-HMF’s toxicity, mutagenicity, and carcinogenicity [12,13]. Therefore, the content of 5-HMF in honey, beer, and coffee has been strictly limited [14,15,16,17]. Despite the previous concern on the dangers of 5-HMF, in recent years a theory for the biological activity of 5-HMF was accepted. It has been reported that 5-HMF has favorable biological effects such as anti-oxidant activity [18], anti-hypoxia [19], and inhibition of sickling of red blood cells [20].

As we know, the mechanism of hypoxic injury contains the process of oxidative stress [21] and 5-HMF can protect hepatocyte cell lines against damage induced by H_2_O_2_ *in vitro* [22]. Hence, it is of great interest to gain insight on its possible molecular mechanism of liver injury *in vivo*. Here, the aim of the present study is to investigate whether 5-HMF may exert hepatoprotective effects on acute alcoholic liver oxidative injury in mice and to further explore the possible mechanisms involved.

## 2. Results and Discussion

### 2.1. Analysis and Isolation of 5-HMF from Schisandra chinensis

**Figure 2:**
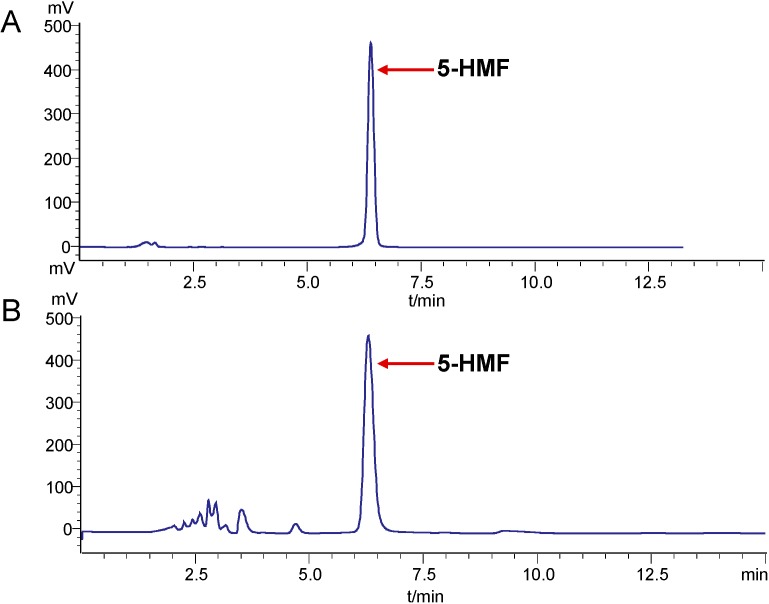
HPLC chromatograms of 5-HMF from the fruits of *S. chinensis*. Reference standard (**A**) and sample (**B**).

The fruits of *S. chinensis* are widely used as a Chinese remedy for liver damage for several centuries [23]. In addition to lignans, the 5-HMF derived from the heat-proceed *S. chinensis* may exhibit hepatoprotective and antioxidant effects [11]. Here, 5-HMF in *S. chinensis* was analyzed by HPLC method. The results showed that the content of 5-HMF is 2.5 ± 0.2 mg/g of dried sample (Figure 2).

### 2.2. Effects of 5-HMF on Body Weight and Organ Index

Body weight is considered as a putative indicator of health. In the present study, the body weight of mice treated with alcohol did not change in comparison with control and 5-HMF groups (Table 1). Organ indexes of the liver and spleen were evaluated in mice. Similar to previous studies, liver and spleen indexed were significantly increased in mice that were exposed to ethanol (*p* < 0.05) compared with control group, but decreased in the 15 and 30 mg/kg of 5-HMF (*p* < 0.05).

**Table 1:** Effects of 5-HMF on body weight and organ index in mice.

Group	Dosage (mg/kg)	Initial Wts (g)	Final Wts (g)	Liver Index (×100, mg·g^−1^)	Spleen Index (×100, mg·g^−1^)
Control	—	22.3 ± 3.1	27.5 ± 2.9	4.31 ± 0.14	0.32 ± 0.05
Alcohol	—	23.3 ± 2.6	28.2 ± 3.1	4.74 ± 0.14 *	0.42 ± 0.03 *
Huganpian	350	22.6 ± 2.1	29.3 ± 2.7	4.38 ± 0.14 ^#^	0.36 ± 0.04 ^#^
5-HMF	7.5	23.1 ± 2.9	27.8 ± 3.3	4.31 ± 0.54	0.40 ± 0.06
	15	22.8 ± 2.6	28.6 ± 3.4	4.48 ± 0.19 ^#^	0.37 ± 0.04 ^#^
	30	23.2 ± 2.7	29.1 ± 3.2	4.51 ± 0.14 ^#^	0.35 ± 0.03 ^#^

Values represent the mean ± S.D., *n* = 8; * *p* < 0.05 *vs.* control group; ^#^ *p* < 0.05 *vs.* alcohol group; Wts: weights.

### 2.3. Effect of 5-HMF on Serum Biochemical Markers

The leakage of AST and ALT in the blood indirectly reflects liver failure caused by ethanol-induced hepatotoxicity [3,24]. As showed in Table 2, the levels of ALT and AST in serum were significantly elevated at 12 h following alcohol administration to mice (*p <* 0.05), indicating liver cell damage and that the alcoholic liver injury model had been established successfully. However, 5-HMF pretreatment for seven days significantly decreased the levels of the serum biochemical indicators by alcohol-induced hepatic damages. Notably, administration of 5-HMF at different doses (7.5 to 30 mg/kg) recovered the impaired liver functions to varying degrees resulting from alcohol-induced toxicity (*p <* 0.05).

Increasing evidence has demonstrated that the steatosis is characterized by the accumulation of TG and TC in liver, and it is one of the significant responses to ethanol consumption [25,26]. Compared with control, the levels of TG, TC, and L-DLC in the alcohol group were elevated significantly (*p <* 0.05). However, the levels of TG, TC, and L-DLC in all 5-HMF groups were reduced and reversed to near control levels (*p <* 0.05). Interestingly, treatment groups had significant dose-dependent effects on alcoholic-induced lipid peroxidation.

**Table 2:** Effects of 5-HMF on serum ALT, AST, TC, TG and L-DLC levels in mice.

Group	Dosage (mg/kg)	ALT (U/L)	AST (U/L)	TC (mM)	TG (mM)	L-DLC (mM)
Control	—	3.91 ± 0.63	12.62 ± 0.78	2.24 ± 0.23	0.38 ± 0.10	0.75 ± 0.15
Alcohol	—	5.32 ± 0.75 *	28.22 ± 0.71 *	3.77 ± 0.32 *	2.15 ± 0.28 *	1.77 ± 0.31 *
Huganpian	350	4.02 ± 0.44 ^#^	21.25 ± 0.72 ^#^	3.54 ± 0.28 ^#^	0.51 ± 0.19 ^#^	1.36 ± 0.21 ^#^
5-HMF	12.5	4.25 ± 0.24 ^#^	24.23 ± 0.55 ^#^	3.32 ± 0.26 ^#^	1.29 ± 0.15 ^#^	1.55 ± 0.32 ^#^
	25	4.21 ± 0.22 ^#^	23.59 ± 0.64 ^#^	3.12 ± 0.22 ^#^	1.12 ± 0.23 ^#^	1.52 ± 0.26 ^#^
	50	4.01 ± 1.02 ^#^	22.45 ± 0.73 ^#^	2.98 ± 0.33 ^#^	0.80 ± 0.11 ^#^	1.43 ± 0.21 ^#^

Values represent the mean ± S.D. (*n* = 8); * *p* < 0.05 *vs.* control group; ^#^ *p* < 0.05 *vs.* alcohol group.

### 2.4. Effect of 5-HMF on Hepatic Biochemical Markers Activities

Oxidative stress is one of the mechanisms of ethanol-induced liver injury. To eliminate oxidative stress, a number of enzymatic and non-enzymatic mechanisms have evolved to protect against reactive oxygen species (ROS) resulting from oxidative stress in alcoholic liver injury [27,28]. These antioxidant enzymes include CAT, GSH-Px, and SOD.

**Figure 3:**
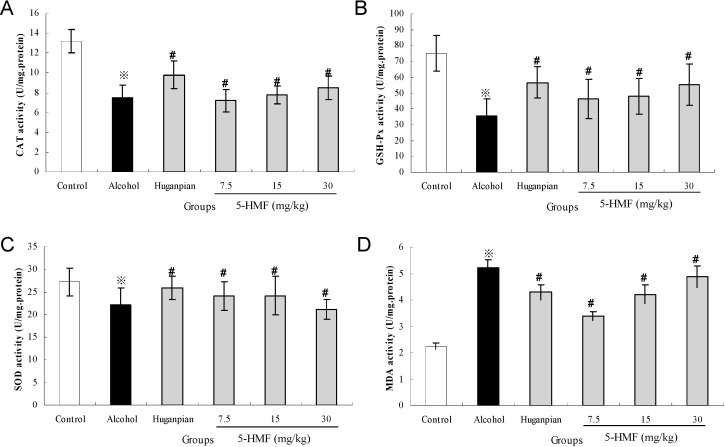
Effects of 5-HMF on hepatic CAT (**A**); GSH-Px (**B**); SOD (**C**) and MDA (**D**) activities in mice. Data represent the mean ± S.D. (*n* = 8); Significant differences were indicated by ^※^ *p* < 0.05 *vs.* control group. ^#^ *p* < 0.05 *vs.* alcohol group.

As shown in Figure 3, compared to the control group, the hepatic antioxidant CAT, GSH-Px and SOD activities in the alcohol group were decreased by 43.2%, 52.7%, and 18.8%, respectively. However, pretreatment with 5-HMF for seven days completely prevented the decrease (*p <* 0.05). MDA is a decomposition product and has been used as a biomarker of lipid peroxidation in liver [4]. On the contrary, compared with that in the normal group, the level of MDA in the alcohol group was elevated by 57.01% (*p <* 0.05). However, the elevation of hepatic MDA concentration clearly decreased in 5-HMF pretreated mice (*p <* 0.05).

### 2.5. Effect of 5-HMF on Hepatic Inflammatory Markers

The activation of TNF-α and IL-1β, the two major inflammatory mediators implicated in inflammation, plays a critical role in the progression of alcoholic liver injury. TNF-α and IL-1β can provoke hepatocellular injury and death [29,30].

**Figure 4:**
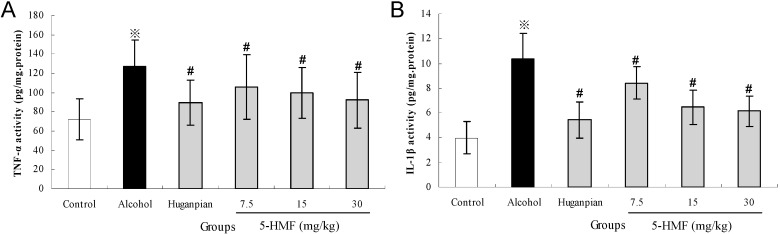
Effects of 5-HMF on hepatic TNF-α (**A**) and IL-1β (**B**) in mice. Data represent the mean ± S.D. *n* = 8; Significant differences were indicated by ^※^ *p* < 0.05 *vs.* control group. ^#^ *p* < 0.05 *vs.* alcohol group.

To investigate the effects of ethanol on the pro-inflammatory response, the expression of TNF-α and IL-1β in liver tissue sections were determined by enzyme-linked immunosorbent assay (ELISA). As indicated in Figure 4, the results revealed considerable up-regulation of hepatic TNF-α and IL-1β after alcohol injection by 43.1% and 61.4%, respectively (*p <* 0.05). Consistent with the results that 5-HMF can prevent TNF α-induced monocytic cell adhesion [31], pretreatment with 5-HMF attenuated TNF-α and IL-1β activity considerably compared with the alcohol group (*p <* 0.05).

### 2.6. Pathological Observations

**Figure 5:**
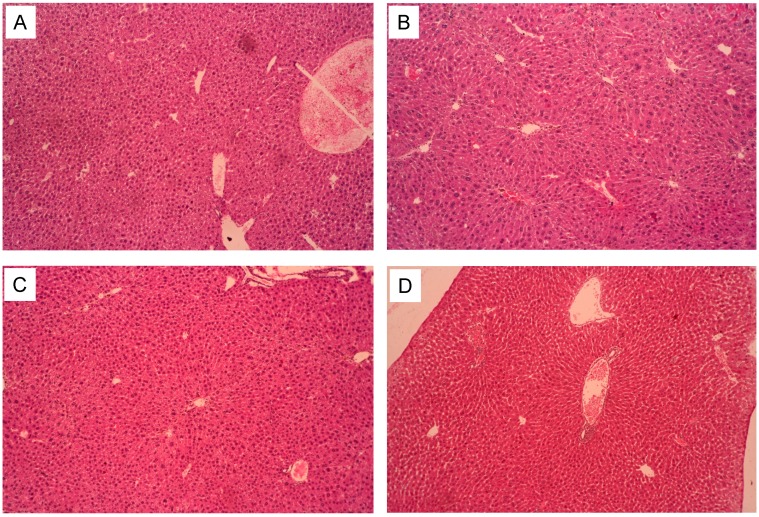
Photomicrographs of liver sections obtained from control group (**A**); alcohol group (**B**); positive control group (**C**) (Huganpian, 350 mg/kg), and 5-HMF (**D**) (30 mg/kg) (magnification, all 100×).

The following figures shows representative photomicrographs of livers obtained from different treatment groups. The normal group had a clear structure of the hepatic lobule and regular hepatic cords with central veins, the cell nucleus was normal and there was no edema, fatty degeneration or visible lesions (Figure 5A). However, in the alcohol group, typical pathological characteristics including necrosis, inflammatory infiltration and extensive vacuolar degeneration confirmed the successful establishment of alcoholic induced liver injury (Figure 5B). Pretreatment of positive drug (Huganpian) and 5-HMF exerted a protective effect against alcohol-induced nuclear damage, whereas the Huganpian group showed only minor hepatocellular necrosis and infiltration of inflammatory cells (Figure 5C). Treatment with high dose 5-HMF (30 mg/kg) before alcohol exposure noticeably attenuated the apoptotic cells and inflammation, almost similar to the normal group (Figure 5D). From the results, we speculate that 5-HMF pretreatment might alleviate ethanol-induced liver damage.

### 2.7. The Liver Pathological Classification and Grading

Systems for grading and staging incorporate the view that necroinflammation is not only a measure of severity but also of ongoing disease activity and the parameter most potentially responsive to therapy [32]. As shown in Table 3, the pathological change in liver is mainly hepatic steatosis and occurs mainly in the central veins. The alcohol group presented a significant liver injury relative to the normal group. The positive group and 5-HMF treatment groups alleviated the steatosis state in different degrees. Huganpin, a liver-protecting agent, is used for preventing and curing the liver injury. In the experiment, the dosage of 350 mg/kg refers to daily human dosage. As a positive drug, Huganpian exerts significant effects on alcohol-induced liver injury.

**Table 3:** Pathological changes in the liver and Ridit analysis.

Groups	Dosage (mg/kg)	*n*	Steatosis Grade					Ridit Analysis
			0	1	2	3	4	
Control	—	8	8	0	0	0	0	0.23
Alcohol	—	8	0	3	4	1	0	0.78 *
Huganpian	350	8	5	2	1	0	0	0.41
5-HMF	7.5	8	2	4	1	1	0	0.59 ^#^
	15	8	3	4	1	0	0	0.51 ^#^
	30	8	4	2	1	1	0	0.49 ^#^

The steatosis stages were classed on the basis of the H&E staining of liver sections. The data were analyzed by Ridit analysis. * *p* < 0.05 *vs.* control group, ^#^ *p* < 0.05 *vs.* alcohol group. Grading standard: 0 = no steatosis, 1 = little steatosis (no more than 1/4), 2 = mild steatosis, (no more than 1/2), 3 = moderate steatosis (no more than 3/4) and 4 = severe steatosis (almost 100%).

## 3. Experimental Section

### 3.1. Chemicals and Reagents

The fruits of *S. chinensis* were collected in Tonghua city and authenticated by Professor Ying-ping Wang, Institute of Special Wild Economic Animals and Plant, CAAS.

Commercial assay kits for alanine aminotransferase (ALT), aspartate transaminase (AST), triglyceride (TG), total cholesterol (TC), Low density lipoprotein cholesterol (L-DLC), catalase (CAT), malondialdehyde (MDA), superoxide dismutase (SOD) and glutathione peroxidase (GSH-Px) were purchased from Nanjing Jiancheng Institute of Biotechnology (Nanjing, China). Huganpian (batch NO. 091103), a liver protectant, mainly contains *Radix bupleuri*, *Oriental wormwood*, *Isatis root*, and *S. chinensis* were purchased form Zhejiang Medicine Co., Ltd. Tumor necrosis factor-α (TNF-α) and interleukin-1receptor (IL-1β) were purchased from R&D System (Minneapolis, MN, USA). HPLC-grade methanol was purchased from Fisher Chemicals (Waltham, MA, USA). Other chemicals, such as alcohol were all of analytical grade from Beijing Chemical Factory, Beijing, China.

### 3.2. Extraction, Isolation and Analysis of 5-HMF

Dry and powdered fruits of *Schisandra chinensis* (200 g) were placed into volumetric flasks with 5 L of methanol added, and sonicated in a water bath at 40 °C for 30 min three times. After the extraction procedure, the filtered solutions were concentrated to dryness under vacuum at 45 °C. The extract was then loaded onto a repeated silica gel column to give a 5-HMF-rich fraction. The above fraction was dissolved in the mobile phase (10% methanol) of semi-preparative liquid chromatography separation to give purified 5-HMF with 202 mg. The structure of 5-HMF was elucidated on the basis of UV, NMR, ESI-MS, retention times of HPLC by comparison of spectral and elemental analyses of standard 5-HMF, and previous reported data.

The 5-HMF in *S. chinensis* was determined by HPLC analysis using a SHIMADZU LC-20AT system with UV detector. The HPLC method employs a Hypersil BDS2 column (250 × 4.6 mm, 5 μm). The column temperature was set at 30 °C and detection wavelength was set at 280 nm. The mobile phase consisted of 10% methanol with flow rate of 1.0 mL/min. The 20 μL of sample solution was directly injected into the chromatographic column manually. HPLC chromatograms are shown in Figure 2.

### 3.3. Animals

Forty-eight male 4-weeks old ICR mice, 20–22 g, were used for the alcoholic induced injury experiment. The animals were provided by the Experimental Animal Holding of Jilin University with a Certificate of Quality No. SCXK (JI) 2011-0004 (Changchun, China). The animals were maintained on standard chow in an air-conditioned room at 25 ± 2 °C with a 12 h dark/light cycle. All the procedures were in strict accordance with Chinese legislation on the use and care of laboratory animals.

### 3.4. Experimental Groups and Treatment

The animals were randomly divided into six groups (*n* = 8 per group), normal control, alcohol control, positive control (Huganpian, 350 mg/kg) and three treatment groups. The treatment groups were administered 5-HMF by gastric intubation for seven consecutive days at doses of 7.5, 15 and 30 mg/kg body weight per day, respectively. Except for positive control, the normal and alcohol groups were administered only 0.9% saline. At day 7, normal control mice were given an equal volume of water and the other mice were intragastrically administered a one-time dose of 50% alcohol (4.8 g/kg) 3 h after final administration.

Then all the mice were kept fasting for 12 h, and subsequently anesthetized with CO_2_. Blood samples were collected by the retrobulbar vessels and allowed to clot for 45 min at room temperature. After standing for 1 h, the serum was separated by centrifugation (1500 rpm, 10 min, and 4 °C) and stored at −20 °C for biochemical analysis.

Alternatively, body weight of the animals was weighed before and after the experiment. At the end of the experimental regimen, the animals in different groups were sacrificed promptly by cervical vertebra dislocation. Livers and spleens were dissected quickly, washed twice with saline, blotted dry on a filter paper, and wet weight were measured. At the same time, the size, appearance, and texture cut surface were recorded as well. A small piece of tissue was cut off from the same part of the left lobe of the liver in each mouse and fixed in 10% buffered formalin solution (*m*/*v*) for histopathological analysis. The remaining liver tissues were stored at −80 °C for hepatic homogenate preparation.

### 3.5. Assay for Serum Biochemical Markers

Serum was used for the spectrophotometric determination of AST, ALT, TC, L-DLC and TG using commercially available diagnostic kits form Nanjing Jiancheng Institute of Biotechnology (Nanjing, China).

### 3.6. Assay for Hepatic Antioxidant Activities and Oxidative Stress Marker

For the antioxidant activity assays, liver tissue was homogenated in 50 mM phosphate buffer (pH 7.4). The resulting suspension was then centrifuged at 13,000× *g* for 15 min at 4 °C, and the supernatant was used for the measurement. Levels of CAT, GSH-Px and SOD in liver homogenates were measured by commercial kits according to the manufacturer’s instructions (Nanjing Jiancheng Institute of Biotechnology). CAT activity was measured in terms of μM of H_2_O_2_ consumed/min/mg protein and it was followed by the method of Claiborne (1985) [33]. GSH-Px activity was monitored according to the method of Mohandas *et al.* [34]. The activity of SOD was measured by the method of Beauchamp and Fridovich [35]. The concentration of malondialdehyde (MDA) was assayed by monitoring thiobarbituric acid reactive substance formation as described by Draper and Hadley [36]. The amount of protein was measured using the Bradford assay [37].

### 3.7. Assay for Inflammatory Markers Activities

Levels of serum TNF-α and IL-1β were determined according to the protocol provided by the manufacture (R&D Systems, Minneapolis, MN, USA). Briefly, adding prepared reagent, samples and standards, antibodies labeled with enzyme, reacting 60 min at 37 °C. After adding stopping solution, measuring and calculation of the OD value was made within 10 min.

### 3.8. Histopathological Examination

For histopathological analysis, the liver tissue (*n* = 8 per group) was fixed in 10% neutral formalin buffer (formalin:phosphate buffer (0.01 M, pH 7.4) = 1:1) for over 24 h, subsequently processed by routine paraffin embedding and sectioned for 5 μm thickness. After hematoxylin-eosin (H&E) staining, slides were observed for histopathological changes using Nikon TE 2000 fluorescence microscope (Nikon, Tokyo, Japan). Representative images were presented. The histopathological characters were used for assessment of histological changes of the liver, including hepatocyte degeneration or necrosis, fatty degeneration, inflammatory cell infiltration and congestion.

### 3.9. Statistical Analysis

All experiments were performed three times independently. Data were presented as means ± S.D. Statistical significance was determined by one-way analysis of variance (ANOVA) followed by least significance difference (LSD) multiple comparison tests using SPSS 16.0 software (SPSS Inc., Chicago, IL, USA). The histopathological data were analyzed by Ridit analysis; *p* < 0.05 or *p* < 0.01 was considered to be significant.

## 4. Conclusions

In this study, it has been clearly shown for the first time that 5-HMF can protect alcohol-induced liver damage as evidenced mainly by the restoration of the enzymatic antioxidants CAT, GSH-Px and SOD, together with lowered serum and liver lipid peroxidation, and reduced TNF-α and IL-1β levels. Moreover, 5-HMF also can ameliorate lipid degradation and hepatocyte apoptosis. Overall, our findings provide substantial evidence that 5-HMF might be beneficial for the potential prophylactic and therapeutic management of the liver injury.
